# Burden and factors associated with post-stroke depression in East central Nigeria

**DOI:** 10.4314/ahs.v17i3.29

**Published:** 2017-09

**Authors:** Sam Chidi Ibeneme, Akachukwu Nwosu, Canice Chukwudi Anyachukwu, Georgian C Ibeneme, Muideen O Bakare, Gerhard Fortwengel, Dnyanesh Limaye

**Affiliations:** 1 Department of Medical Rehabilitation, Faculty of Health Sciences, College of Medicine, University of Nigeria, Enugu Campus, Enugu, Nigeria; 2 National Institute for Sports, Surulere, Lagos, Nigeria; 3 Ebonyi State University, Department of Nursing Sciences, Abakaliki, Nigeria; 4 Department of Adult and Paediatric Psychiatry. Federal Neuro-Psychiatry Hospital, New Haven, Enugu; 5 Hochschule Hannover - University of Applied Sciences and Arts Fakultät III, Expo Plaza 12, Hannover, Germany

**Keywords:** Symptoms of post-stroke depression, modifiable characteristics of the vulnerable patients, African socio-cultural context

## Abstract

**Objective:**

To determine the burden and factors associated with post-stroke depression in East central Nigeria.

**Method:**

We carried out this cross-sectional study of 50 stroke survivors (mean age=54.8 ± 8.8 years), at the physiotherapy Department of the University of Nigeria Teaching Hospital, Enugu. Data were collected using Becks Depression Inventory , it was analyzed using Z-scores, Chi-square test and univariate logistic regression.

**Results:**

PSD was more common in females (45.45%); middle-age(60%) adults(27–36/47–56 years respectively); living with spouse (45%); left cerebral lesions (40.74%). Self-employed and unemployed (66.67%), respectively. Age was significantly associated with depression (p=0.03), and was related to the risk ofOR3.7 (95% CI 1.1–12.0 )

**Conclusion:**

Age could be a risk factor for PSD, which was more prevalent in the elderly than young/middle-age adults, female gender, left cerebral lesion, complications, cold case; those living with a spouse, self-employed and unemployed.

## Introduction

An insight into the link between the biological, social, and interpretive orientations that determines an individual's emotional behavior, elucidates the basis for likely differences in emotional expression in socio-cultural contexts^1^. This may have implications for etiopathology and treatment of mood disorders, including post-stroke depression (PSD). Therefore, determining the distribution of symptoms PSD in an African population in relation to some predisposing factors may help to identify the modifiable characteristics of vulnerable individuals that may be peculiar to an African socio-cultural context, which may be targeted to enhance recovery from stroke.

Depression is a negative variable that most often limits the rate of recovery during rehabilitation of stroke patients. In fact, the occurrence of post-stroke depression (PSD) has been associated with poorer treatment outcomes and increased the length of stay in hospital^2^. Importantly, the occurrence or persistence of PSD >3 years after stroke can impede the process of rehabilitation^2^. Thus, understanding the distribution of the symptoms of PSD in relation to some predisposing factors may help to identify the modifiable variables likely to influence the psyche of the vulnerable individuals. However, most studies have examined these variables without consideration for the varied socio-cultural influences. For instance, an understanding of the modifiable variables that may be peculiar to an African rather than Western socio-cultural context will be more relevant in effectively targeting vulnerable individuals to enhance recovery from PSD among Physiotherapy patients of African origin, and vice versa. Among other benefits, this could translate to improved treatment outcomes, shorter length of stay in the hospital, unimpeded rehabilitation process and reduced cost of treatment for affected individuals.

Several studies have attributed PSD to the location of a causative lesion^2^. Recently, an MRI-based study demonstrated that PSD is associated with lesions within the prefrontal-subcortical circuits^3^. However, there are mixed scientific findings on this matter^4^,^5^, hence, results from another study suggest that several factors extraneous to the neurobiological vectors may be contributory to PSD^6^, including psycho-social variables. In fact, Carney and Freedland^7^ elucidated the mechanistic processes by which “psychological distress (negative mood states or neuroticism or negative affectivity)” relates with a stroke that may be important in defining or even determining the course and prognosis of functional recovery in stroke. This evidence suggests that various types of stressors influence the human psyche, and may derive from the environment and patterns of socialization. Invariably, a modifiable risk factor in Europe may be perceived differently in Africa, and vice versa; and will have different implications in the rehabilitation of patients with stroke in both societies.

PSD may evoke feelings of low self-esteem, guilt, and self-reproach, limitation of interpersonal contact/social integration and somatic symptoms such as eating and sleep disorders.^7^ Therefore, the personality traits that may interact with stroke and trigger PSD in an African population, may vary from what obtains in Europe. In addition, the homeostatic dynamics of the cultural ecosystem of the African extended family system that provides robust social support for stroke survivors may be at variance with experiences in other societies of the world. In essence, a better management of PSD requires identification of the predisposing factors that must be modified/targeted to enhance recovery. Thus, this study sought to:

- determine the distribution of symptoms of post-stroke depression (PSD) in relation to some predisposing factors in African socio-cultural context.
- Identify modifiable characteristics of individuals vulnerable to symptoms of PSD in African socio-cultural context.
- Predict whether stroke survivors have symptoms of PSD based on observed characteristics

These objectives were investigated in the context of personality factors and the severity of the negative physical, psychological, and social consequences of stroke,^8^ which vary from the Western to sub-Saharan Africa societies.

## Materials and method

### Research design

A cross-sectional observational study^9^ involving multiple stroke survivors was conducted

### Area of study

**Setting:** This study was carried out at the outpatient clinic (Adult Neurology Unit) of the Physiotherapy department of the University of Nigeria Teaching Hospital, (UNTH) Ituku-Ozalla. The UNTH is a 600-bed foremost reference hospital established to care for patients from the the East Central state of Nigeria, initially comprising the South East and South-South regions of the country. Presently, the hospital attends to patients from the 5 states within its catchment area. The Physiotherapy out-patient clinic consists of 8 units, and the Physiotherapy department attends to an average of 70 in- and out-patients, daily.

### Subjects selection

Fifty stroke survivors (22 females and 28 males) were selected by convenience sampling method and evaluated based on case presentations such as etiology, anatomic locations of the lesion, age, gender, employment status, marital status, the presence of complications and duration of stroke. The subjects comprised only those that gave their informed consent and were available at the time of the study, which lasted for 6 months.

### Selection criteria

#### Inclusion criteria

- Stroke survivors must be 26 – 66 years old.
- Stroke survivors included only those that receive physiotherapy treatment at the University of Nigeria Teaching Hospital, Ituku/Ozalla
- Stroke survivors included those who had a stroke for 1–3 years, and > 3 years, respectively.

#### Exclusion criteria

- Stroke survivors unable to communicate meaningfully (because of severe aphasia or cognitive dysfunction.
- Stroke survivors with TIA (transient ischemic accident
- Stroke survivors with other pathologies of neurological or orthopedic nature.

### Research instruments

The test instruments include the Modified motor assessment scale, Mini-mental Motor Examination and Beck's depression Inventory. Each instrument was translated to from English to the Igbo language by an expert and back-translated from Igbo to English by another expert to ensure they retained their meaning.

### Modified motor assessment scale

Modified motor assessment scale is a reliable measure of motor recovery for patients with stroke. This scale was used to monitor recovery and measure the motor function of the patients with tasks that are frequently the focus of rehabilitation during stroke management. “It is an 8-task instrument and includes measures for rolling, sits up over the bed edge, sits to stand, walking, and advanced hand activities, among others”^11^. Items on the Modified Motor Assessment Scale are scored on a scale of 0 – 6, where 0 indicates “completely dependent” and 6 denotes “independent, efficient level of function”. Intra-rater reliability of the instrument is 0.83 to 1.00 with a median of 0.97.

### Mini-Mental State Examination (MMSE)

The MMSE is a brief screening tool to provide a quantitative assessment of cognitive impairment and to record cognitive changes over time. A possible score of 30 is utilized to provide insight on an individual's current cognitive performance based on direct observation of completion of test items or tasks. Generally, this study applied a conservative score of <27 as the accepted cutoff indicating the presence of cognitive impairment which was also used in a previous study involving patients with stroke.^12^

### Beck's Depression Inventory (BDI)

BDI is used for assessing recovery and efficacy of interventions designed to relieve depression. A 21-item self-report multiple choice questionnaire (Becks Depression Inventory, BDI) which assesses mood, pessimism, and sense of failure, self-dissatisfaction, guilt, punishment, self-dislike, self-accusation, social withdrawal, work difficulties, insomnia and so on, was also used in this study. Items 1–3 assess symptoms that are psychological in nature, while items 4 – 21 assess more physical symptoms. These were scored on a scale of 1–30 as follows:

1 – 10: Normal
11 – 16: Mild mood disturbance
17 – 20: Borderline clinical depression
21 – 30: Moderate depression
>30: Severe depression

Beck's Depression inventory was originally designed as a tool for quantitative assessment of the intensity or depth of the depression. Consequently, it is useful in monitoring progressive changes in depression over time and served as an objective measure of depression in this study. BDI has been used extensively in Nigeria, and the instrument validated among Nigerian youths^13^. Internal consistency studies demonstrated a correlation coefficient of 0.86 for the test items, and the Spearman-Brown correlation for the reliability of the BDI yielded a coefficient of 0.93^14^. “At a cut-off score of 18 and above, the Becks Depression Inventory has a sensitivity of 0.91, specificity of 0.97, positive predictive value (PPV) of 0.88 and negative predictive value (NPV) of 0.98”^13^. Content validity would seem to be quite high since the BDI appears to evaluate well, a wide variety of symptoms and attitudes associated with depression. Beck reports a study in which coefficients of 0.65 and 0.67 were obtained in comparing results of BDI with psychiatric ratings of patients^14^. Other instruments used in this study include:

- Stopwatch, which was used to record the time during the motor assessment scale measurement.
- Comb, spoon, tea cups, and a jelly box or precision box.

### Procedure for data collection

The researchers visited the Physiotherapy out-patient clinic at the UNTH 3 times weekly on appointment days set aside for treatment of stroke survivors after the institutional board had approved the study protocol. All the patients gave their written informed consent to participate in the study after the purpose was explained to them. The patients were reviewed with respect to etiology, anatomic locations, age, gender, employment status, marital status, the presence of complications and duration of the stroke. Thereafter, patients that met the selection criteria were assessed with the Mini-Mental Status Examination tool to check for intact cognition. Also, each stroke survivor was assessed with the modified motor assessment scale to determine the level of motor functioning impairment. Subsequently, the BDI was completed by the patients; however the researcher provided assistance in filling the subjects' choices for each item in a few instances where the patients were unable to write due to motor dysfunction, so far the stroke survivor(s) had intact cognition and ability to comprehend the item questions. At the end of the study, the data recorded were retrieved and tabulated for analysis.

In this study, the duration of stroke was identified as a predisposing factor, which was classified into recent and cold cases. A recent case was operationally defined as a case with 1–3 years of onset while a cold case is one that occurred >3 years ago. Marital status was operationalized as living with or without spouses, and assessed as a predisposing factor for depression. In the context of this study, those living without their spouses included divorcees, single, separated, and widowed that had no companions. Employment status of the patients was assessed as a predisposing factor for depression in stroke. In the context of this study, three categories were considered, namely, unemployment, self-employment and paid employment. Housewives were categorized as unemployed, while individuals who were self-employed were operationalized to include those that worked for themselves without socio-economic safety nets, such as Artisans, Drivers, Farmers, and Petty traders, whereas those categorised as being on “paid employment” included those that were employed and paid by established businesses with socio-economic safety nets, such as civil servants, bankers, and retirees

### Data analyses

The data, collected for this study, were analyzed using Graph Pad Prism software. The results were presented in tables. Inferential statistics was utilized including Z-test to determine proportional significance, and chi-square was applied to determine the level of association between the variables of interest. Univariate logistical regression analysis was used in a stepwise fashion to assess variables that were independent predictors of PSD. All Prisms computes the confidence interval of the odds ratio and relative risk using approximations detailed by Altman^15^. If any cell has a zero, Prism adds 0.5 to all cells before calculating the relative risk, odds ratio, or P1-P2 (to prevent division by zero). Alpha was set at 0.05.

**Ethical approval:** Ethical approval from the Health Research Ethics Committee of the University of Nigeria Teaching Hospital, Ituku/Ozalla, was obtained prior to the study. All the patients gave their written informed consent to participate in the study after the purpose was explained to them. Participants were assured of the confidentiality of the information they provided and anonymity of the source of information. It was made clear to the participants that they had the right to refuse to participate or to withdraw at any stage of the project, and these rights were respected all through the research process as stipulated in the Helsinki declarations.

## Results

Age distribution of the sample showed that most of the patients were aged 47–56 years with the least being those aged 27–36 years. Distribution of the symptoms of PSD based on age range, duration of the stroke, living with a spouse, type of employment, gender, anatomic location of cerebral lesions and complications, showed varied outcomes (tables 1–2), respectively.

**Table 1:** Distribution of depression in relation to some socio-demographic characteristics of respondents (N=50)

Socio-demographic	N=50	Non-Depressed N (%)	Depressed N (%)
**Sex**			
Female	22	12(54.55)	10 (45.46)
Male	28	18(64.29)	10 (37.71)
**Age Range**			
27–36	2	0(0)	2 (100)
37–46	6	3(50)	3 (50)
47–56	15	6(40)	9 (60)
57–66	27	19(70.37)	8 (29.63)
**Marital**			
**Status**			
LWS	40	22(55)	18 (45)
NLWS	10	7(70)	3 (30)
**Employment**			
**status**			
SE	9	4(44.44)	5 (66.67)
PE	38	23(60.53)	15 (39.47)
UE	3	1(33.33)	2 (66.67)

LWS = living with spouse; NLWS = Not living with spouse; SE = Self-Employed; PE = Paid Employement, UE = Unemployed

**Table 2:** Distribution of depression in relation to some anatomic and clinical variables (N=50)

Variables	Non depressed N (%)	Depressed N (%)	Total N=50	χ^2^	*p*-value
Location of Cerebral Lesion					
Left	16(59.26)	11 (40.74)	27	0.01	0.91
Right	14(60.87)	9 (39.13)	23		
Total	30	20	50		
Complications					
(Dementia &				3.46	0.18
Hypertension)	22(55)	18 (45)	40		
Nil	8(80)	2 (20)	10		
Total	30	20	50		
Duration of					
Occurrence (years)					
1–3	21(63.64)	12 (36.37)	33	2.28	0.13
.3	9(52.94)	8 (47.06)	17		
total	30	20	50		

For instance, the distribution of the symptoms of PSD based on age range (table 1) showed that among those aged 27–36 years, 37–46 years, 47–56 years and 57–66 years, symptoms of PSD was prevalent in 100%, 50%, 60%, and 29.63%, of the population, respectively. Furthermore, in stroke survivors with <3 years of onset (recent cases), 36.37% of the cases had symptoms of PSD while 47.06% of those with >3 years of the onset of stroke (cold cases), had symptoms of PSD. Similarly, 45% of stroke survivors living with a spouse and 30 % of those living without a spouse, had symptoms of PSD. Furthermore, the majority (66.67%) of stroke survivors who were self-employed and (66.67%) unemployed had symptoms of PSD, respectively, whereas only less than half (39.47%) of those in “paid employment” had symptoms of PSD.

Meanwhile, 45.46% of the female stroke survivors had symptoms of PSD compared to 35.71% recorded among their male counterparts. Symptoms of PSD were also observed in 45% of the stroke survivors with complications compared to 20% recorded for those without complications (Table 2). In addition, 40.74% of stroke survivors with left cerebral lesion had symptoms of depression compared to 39.13% recorded for those with right cerebral lesion. Statistical analysis of the results showed that there was no significant association between symptoms of depression and: duration of stroke(χ2 = 0.53, p = 0.33), living with spouse(χ2 =3.93, p = 0.27)), type of employment(χ2 =7.32, , p = 0.58) ), gender(χ2 = 0.49, p = 0.34), location of cerebral lesion(χ2 = 0.01, , p =0.91), and complications (χ2 = 0.01, , *p* = 0.14), respectively. In contrast, there was a significant association between symptoms of depression and age(χ2 = 4.92, , *p* = 0.03), The results of the multivariate analysis showed that the following variables were less likely to be associated woth PSD, namely: location of cerebral lesion [OR1.1 (95% CI 0-3-3-33, *P* > 0.92, φ = +0.31, φ^2^ = 0.1], presence of complications [3.2(0.6-17-4, *P* φ = 0.13, φ = −0.2, φ^2^ = 0.04], duration of stroke [0.6 (0.2-2-1, *P* = 0.33, φ = −0.02, φ^2^ = 0.0004], marital status [2.5 (0.6-10-4, *P* = 0.18, φ = −0.17, φ^2^ = 0.0289], employment status [1.1 (0.3–4.1, *P* = 0.58, φ = +0.02, φ^2^ = 0.0004], and gender [0.7 (0.2-2-1, *P* = 0.34, φ = +0.2, φ^2^ n= 0.04)]. In contrast, age was more likely to be associated with PSD [3.7 (1.1-12.0, *P* = 0-03*, φ = +0.31, φ^2^ = 0.1)].

## Discussion

Our study revealed that a reasonable proportion of stroke survivors responded to their adversity with symptoms of depression, and support a previous view that strokes “should also be considered a negative life event to which patients may respond to with symptoms of depression.”^16^ This may translate to depressed bodily functions with negative implications for rehabilitation and functional recovery of affected survivors. It was further revealed that more than half of stroke survivors that were self-employed, unemployed and of ages 27–36 years, 37- 46 years, and 45–46 years, had symptoms of PSD. Thus, in the context of African socio-cultural diversity, some stroke survivors in the above age groups and status of employment may have predominantly negative interactions with the variables of socialization that may predispose to symptoms of PSD, respectively. This is especially true for age which was significantly associated with symptoms of PSD and identified as a major risk factor for depression in stroke survivors.

## Relevance of findings to the field

Profiling each modifiable risk factor revealed identifiable trends that may have socio-cultural resonance. Nevertheless, divergent views were expressed in the literature on the nature of the relationship between some of these risk factors (anatomic/clinical/demographic variables) and the PSD. For instance, three major scientific opinions have been crystallized from the literature on the relationship between the location of lesion and PSD, including that: i. there is an association between the right cerebral hemisphere and PSD^17^ ii. the Left cerebral hemisphere has a significant relationship with PSD,^18^ and iii. there is no relationship between the anatomic location of cerebral lesions and PSD^19^. The literature further revealed that even among those with the left cerebral lesion, there was severe depression with those diagnosed with the left anterior cerebral lesion and less severe PSD was related to left posterior lesions.^18^,^20^,^21^ Though this study revealed no significant association between the location of the cerebral lesion and symptoms of PSD, there was a dominant trend characterized by the prevalence of the symptoms of PSD in most of those with left rather than right cerebral lesions. It will be interesting to see if future studies will investigate whether the left, rather than right cerebral lesions, interacts more negatively with socio-cultural parameters in an African population to trigger PSD.

Though there was no significant association between gender and symptoms of PSD, the dominant trend was that majority of the women had symptoms of PSD compared to men. It was further observed that marital status (living with/without a spouse) was not significantly associated with the symptoms of PSD in this study; however, the dominant trend suggests that stroke survivors living with a spouse were more predisposed to symptoms of PSD than those living without a spouse. In the context of various socio-cultural practices, distribution of symptoms of PSD in relation to marital status may have some implications. Also, there was a dominant pattern of prevalence of symptoms of PSD among stroke survivors who had other complications compared to those who do not have such complications. Nevertheless, analyses of the results suggest that the symptoms of PSD were not dependent on the presence of other associated complications or co-morbidities. It was equally revealed that the symptoms of PSD were not dependent on the duration of the stroke, but were higher in cold cases than recent cases. Interestingly, broad ranges of scientific opinion, found in the literature, were multi-directional on this matter. For instance, it appears that whereas a study^20^ reported the high and stable prevalence of PSD in patients with stroke, yet there were changes in patients' depression profile. In the same study,^20^ improvement was noted in 100% of patients diagnosed with major depression within two years after stroke while patients diagnosed with dysthymic depression recorded 30% rate of improvement within the same two-year period.

Remarkably, patients without depressive symptoms developed mood disorders within two years of onset of stroke. A similar study^22^ reported depressive symptoms in 17% of the stroke survivors within the 1st year of onset, and yet improvement/recovery was recorded after the 1st year in those who were earlier diagnosed with early depressive symptoms within three months of onset of stroke. This implied that early onset of PSD in stroke survivors could be improved while those previously undiagnosed with PSD were likely to have depressive symptoms or mood disorders as the duration of stroke increased.

Divergent views on this matter, in relation to research design, have been found in the literature. Thus, whereas a hospital-based study reported a lower PSD in survivors, from 6 months to 3 years after the onset of stroke,^23^ another hospital-based study revealed a decrease in the prevalence of PSD in the 1st year, and a subsequent rise in 2nd and 3rd year, after the onset of stroke.^24^ The same pattern of development was revealed by a rehabilitation-based study design,^25^ Lack of consensus in scientific opinion on this issue was also evident when the assessment instrument is considered. For instance, assessment for PSD in stroke survivors using Becks Depression Inventory (BDI) revealed no change in the PSD symptoms within 12–15 months after the stroke.^26^ This study using the same assessment criteria (BDI) revealed that there were still depressive symptoms in about 36.37% of the patients within 12–36 months of stroke onset, whereas 47.02% of the patients had PSD beyond 3 years of initial onset of stroke. In essence, there was increasing prevalence of PSD as post-stroke duration progressed. This does not agree with an earlier observation that there was a decreasing prevalence of PSD among stroke survivors from the 6th month , as was reported in another study^27^ using the same assessment criteria (BDI).

From the available literature, early onset of PSD in stroke survivors have been evident,^28^,^29^ but a common consensus that delineates the course of depression remains elusive and may highlight the influence of psycho-social variables that predispose to depression in each context. In addition, other possible explanations for the observed trend in literature is that the stroke survivors gradually tend to realize and align with the reality of the loss of function, and as such, PSD assumes a similar course as it also gradually develops and intensifies. Over time, the depressive symptoms seemed more prevalent as may be influenced by failure to realize expected recovery of function following physiotherapy rehabilitation. The robust social support from extended family members, which underlies the continuing importance of kinship network in Africa societies, such as Nigeria, may be the reason that prevalence rate in cold cases was not higher, as reported in this study. This study did not identify any association between the status of employment and symptoms of PSD among stroke survivors, but there was a clear dominant pattern with (66.67%) a higher prevalence of the symptoms of PSD in the self-employed and unemployed, respectively.

## Strength and limitations of the study

This study has some limitations that must be highlighted to ensure that the outcomes are interpreted in the appropriate context. For instance, an exact definitive diagnosis of stroke in the stroke survivors was not made using a CT-scan, thus, there was a possibility of misdiagnosis, and the inclusion of other neurological conditions with similar clinical presentations as stroke. Nevertheless, we relied on the clinical differential diagnosis of stroke, which is equally valid. Also, a self-rated instrument for depression diagnosis is known to have limitations. For instance, it has been suggested that depression influences and skews self-rated personality scores to falsely reflect more pathology, 31 however, another study^32^, presented a contrasting view. Furthermore, there was no significant association between demographic variables and PSD symptoms, which might be due to the lack of power of the statistical tests in view of the small number (n=50) of the participants. Thus, the sample size could be a limitation of the study. Unfortunately, we enrolled all the patients that met the selection criteria and were willing to participate in the study. Though the data analyses did not show significant differences in some cases, nevertheless, it highlighted important trends that may have prognostic value in clinical practice. Therefore, despite these limitations, the objectives of the study were realized to a reasonable extent.

## Implications for care teams and policymakers

The positive association between age and the symptoms of depression suggests that depression is likely to occur with aging, and therefore the elderly stroke survivors should be more vulnerable than the young adults. Nevertheless, it is noteworthy that all the young adults (aged 27 – 36 years) in this study, reported symptoms of depression, and should be studied further. Apparently, the middle-aged adults seemed to handle adverse challenges of stroke better than the rest, hence the least prevalence of the symptoms of depression in their population unlike the young adults who seemed to have the worst coping strategy, hence the highest prevalence of depression in their population. Meanwhile, the fact that depression in patients might also be determined by the interaction between personality factors, and the severity of the negative physical, psychological, and social consequences of stroke,^30^ suggest that patients' perception of the “severity” of these variables may be a relevant determinant of how they react with depression to stroke. In essence, different age groups may react to stroke as much as they perceive it to be an adversity. The fact that all the young adults (aged 27–56 years) had symptoms of depression may suggest that they might have perceived stroke most negatively compared to other age groups, with regards to its physical, psychological and social consequences. This view may explain a previous observation that younger stroke survivors were more likely to become depressed than older stroke victims.^22^,^23^ However, statistical analyses of our findings suggest that the older the stroke survivor, the higher the likelihood of responding to stroke with depression.

## Conclusion

An overview of divergent reports on the scope of depression in stroke survivors from various studies seems to vary depending on patients' clinical characteristics, time of assessment, type of assessment instrument/criteria, population characteristics, study design and definition of depression among others. Therefore the interpretation of such reports must take cognizance of contextual peculiarities of each study as a basis for meaningful application of its findings. This may explain why some studies may not agree with our findings. Nevertheless, the results of our study suggest that the likelihood of responding to stroke with depression was significantly higher among the elderly stroke survivors than any other age group. This implies that the elderly stroke survivors may be targeted from the onset of stroke as the age group that is most vulnerable or at the greatest risk for PSD in some African populations.
